# Characteristics of children of the Microcephaly Epidemic Research Group Pediatric Cohort who developed postnatal microcephaly

**DOI:** 10.1038/s41598-022-19389-w

**Published:** 2022-09-22

**Authors:** Regina Coeli Ferreira Ramos, Demócrito de Barros Miranda-Filho, Celina Maria Turchi Martelli, Thália Velho Barreto de Araújo, Maria Angela Wanderley Rocha, Vanessa van der Linden, Maria Durce Costa Gomes de Carvalho, Laura Cunha Rodrigues, Ulisses Ramos Montarroyos, Wayner Vieira de Souza, Maria de Fátima Pessoa Militão de Albuquerque, Elizabeth B. Brickley, Ricardo Arraes de Alencar Ximenes

**Affiliations:** 1 Departamento de Infectologia Pediátrica, Hospital Universitário Oswaldo Cruz, Recife, Brazil; 2 Pós-Graduação em Ciências da Saúde, Universidade de Pernambuco, Recife, Brazil; 3 Instituto Aggeu Magalhães, Fiocruz, Recife, PE Brazil; 4 Programa de Pós-Graduação em Saúde Coletiva, Universidade Federal de Pernambuco, Recife, Brazil; 5 Faculdade de Ciências Médicas, Universidade de Pernambuco, Recife, Brazil; 6 Hospital Barão de Lucena, Recife, Brazil; 7 Department of Infectious Disease Epidemiology, London School of Hygiene & Tropical Medicine, London, UK; 8 Departamento de Saúde Coletiva, Instituto Aggeu Magalhães, Fiocruz, Recife, Brazil; 9 Universidade Federal de Pernambuco and Universidade de Pernambuco, Recife, Brazil

**Keywords:** Diseases, Medical research, Signs and symptoms

## Abstract

The number of studies published on postnatal microcephaly in children with Congenital Zika Syndrome is small, clinical presentations vary and aspects of the evolution of these children remain unclarified. The present case series examined clinical characteristics and assessed the growth velocity of the head circumference, weight and height Z-scores in 23 children who developed postnatal microcephaly during follow-up in the Microcephaly Epidemic Research Group Pediatric Cohort. To estimate the change in the head circumference, weight and height Z-scores over time and compare the mean difference between sexes, we used multilevel mixed-effects linear regressions with child-specific random effects. Among these children, 60.9% (n = 14/23) presented with craniofacial disproportion, 60.9% (n = 14/23) with strabismus, 47.8% (n = 11/23) with early onset seizures, 47.8% (n = 11/23) with dysphagia and 43.5% (n = 10/23) with arthrogryposis. Of the 82.7% (n = 19/23) children who underwent neuroimaging, 78.9% (n = 15/19) presented with alterations in the central nervous system. Monthly growth velocity, expressed in Z-scores, of the head circumference was − 0.098 (95% CI % − 0.117 to − 0.080), of weight was: − 0.010 (95%-CI − 0.033 to 0.014) and of height was: − 0.023 (95%-CI − 0.046 to 0.0001). Postnatal microcephaly occurred mainly in children who had already presented with signs of severe brain damage at birth; there was variability in weight and height development, with no set pattern.

## Introduction

Microcephaly is the most characteristic component of Congenital Zika Syndrome (CZS), likely due to the presence of craniofacial disproportion^1–3^. Other clinical features may also be involved in CZS, such as ophthalmologic abnormalities, including retinal and macular changes, strabismus, or microphthalmia^4^, in addition to neurological and musculoskeletal changes in newborns, such as arthrogryposis^5,6^. The most frequently observed abnormalities in the central nervous system (CNS) are intracranial calcifications, malformations of cortical development, a reduced volume of the cerebral white and gray matter, and ventriculomegaly^5,6^. Although the most severe cases of CNS involvement are already well known, the phenotypic spectrum of CZS has not been fully defined and less severe cases, with minor repercussions on the CNS, have been described. There are reports in the literature of children with prenatal Zika virus (ZIKV) exposure who presented with late-onset microcephaly^5,7–12^, thereby justifying the need for clinical follow-up for early identification and intervention^13,14^. However, the number of studies investigating postnatal microcephaly is small, clinical presentations vary and some aspects of the evolution of these children need to be better understood and characterized.

Although head circumference (HC) measurement is recommended for individual follow-up from 0 to 24 months, weight and height are accepted as the most important parameters in relation to the assessment of child growth^15^. Weight may be influenced by several factors while length is considered a more reliable indicator. Variation in length in the first year of life is largely influenced by perinatal factors (e.g., maternal age, alcohol use, prematurity), but after 12 months, determination is predominantly genetic. However, other factors like structural damage to the CNS of children, as it occurs in children with congenital Zika-related microcephaly, may affect their weight and height development through different mechanisms (e.g., altered feeding patterns, high rates of dysphagia, and endocrine disorders)^16–18^.

The present study assessed the growth velocity of the HC, weight and height Z-scores among children born during the 2015–2017 microcephaly epidemic in Pernambuco, Brazil, who presented at birth with appropriate HC for gestational age (GA) and sex, according to the International Fetal and Newborn Growth Consortium for the 21st Century (INTERGROWTH-21st) curves^19^ but later developed microcephaly.

## Methods

This is a case series of children who developed postnatal Zika-related microcephaly and were followed-up as part of the Microcephaly Epidemic Research Group Pediatric Cohort (MERG-PC)^20^.

For this study, we defined microcephaly at birth as a HC of at least 2 standard deviations (SD) below the mean for GA and sex, according to INTERGROWTH-21st curves^19^, and in the follow-up, according to the World Health Organization (WHO) Infant Growth Standards^12,21^. The definition of CZS was based on the criteria of França et al.^22^: confirmed cases were defined as those with laboratory evidence for ZIKV infection (maternal reverse transcription polymerase chain reaction [RT-PCR] or serology during pregnancy and/or newborn positive for ZIKV Immuglobulin (Ig)M in the cerebrospinal fluid [CSF]), independently of other findings. Probable cases of CZS were classified based on neuroimaging, and negative laboratory results for ZIKV and for other congenital infections (cytomegalovirus, toxoplasmosis, syphilis, and human immunodeficiency virus [HIV]). Possible cases presented with characteristic neuroimaging findings for CZS (computed tomography (CT), magnetic resonance imaging (MRI) or transfontanelar ultrasound [TFUS]), no laboratory evidence of ZIKV infection and without results for one or more of the three infections (syphilis, toxoplasmosis, and cytomegalovirus)^22^. HC was measured with an inelastic measuring tape positioned over the occipital prominence and the arch of the eyebrow. With the child's head fixed, the tape was placed firmly around the frontal bone over the supraorbital sulcus and then wrapped around the head, at the same level on each side, over the maximal occipital prominence^23,24^. Term births were defined as those occurring with 37 or more weeks of GA. Birth weight was assessed and classified as appropriate, small or large for GA and sex (AGA, SGA and LGA respectively), using the INTERGROWTH-21st standards^19,25–28^. For prospective follow-up from birth up to 36 months of age, repeated HC, weight and length measurements were performed according to the routine protocol (20), in addition to brain imaging when indicated. Z-scores for HC, weight, length, and body mass index (BMI) were calculated using the WHO anthropometric calculator, the WHO Anthro 3.2.2^29^. Microcephaly cases were classified as severe when the HC Z-score was ≤  − 3 SD of the growth curves established according to sex and GA, and moderate when the HC Z-score was ≤  − 2 and >  − 3 SD. Child's length was measured with a horizontal stadiometer. Weight was assessed on a calibrated precision digital platform scale. As children were unable to remain in an upright position without assistance, they were assessed in terms of the following weight difference: weight of adult assistant plus child weight minus adult assistant weight. The BMI was calculated by dividing weight in kilograms by squared height in meters and assessed against the WHO Child Growth Standards^30^. Ophthalmologic assessment was conducted through study of the retina. All selected children presented a HC at birth that did not exceed 2SD below the mean for GA and sex, and therefore did not meet the definition of microcephaly at birth (at least 2SD below the mean, by the-Z score)^29^.

Statistical analysis was performed using STATA SE 14.2 (College Station, TX, USA). Categorical variables were summarized in absolute numbers and percentages. Continuous variables were summarized as means and standard deviations. To estimate the change in HC (Z-score), weight (Z-score) and height (Z-score over time) we used multilevel mixed-effects linear regressions with child-specific random effects. To compare the mean Z-score difference between groups over time we used mixed effects random intercept regression.

The Research Ethics Committee of the Hospital Universitário Oswaldo Cruz approved the study on October 10, 2016 (Ethical Clearance Certificate—CAAE 52803316.8.0000.5192). The children's legal guardians signed an informed consent form to participate in the study. All research was conducted in accordance with the Declaration of Helsinki and Brazil's codes and regulations regarding research on human subjects.

## Results

In the MERG-PC, 23 children who presented at birth with a HC > 2SD (mean (SD) for HC Z-score = − 1.09 (0.95)) later developed postnatal microcephaly (Table 1). These children were prospectively followed up during a median of 4 visits (P_25_–P_75_: 3 to 5, range: 2 to 8) with the pediatric infectious disease division. Of the 23 children with late-onset microcephaly, the HC at birth ranged between 32 and 33 cm, and 9 (39.2%) children were female. Seventeen (73.9%) of the children were born appropriate for gestational age (AGA), 2 (8.7%%) were born small for gestational age (SGA), and 1 (4.3%) was born large for gestational age (LGA). Of the 20 with available information on gestational age at delivery, 100% were born at term (37–41 weeks of gestation). Positive results for ZIKV were obtained in 8 of the 11 (72.7%) children whose CSF was tested at birth by IgM Antibody Capture Enzyme Linked Immunosorbent Assay (MAC-ELISA).

**Table 1:** Mean Z-score at birth and monthly change in head circumference, weight and length of children who developed postnatal microcephaly in the Microcephaly Epidemic Research Group Pediatric Cohort (MERG-PC), in Pernambuco, Brazil.

Groups	Mean Z-score at birth (95% CI)	Mean monthly change in Z-score (95% CI)	p-value
**Head circumference Z-score**			
All	− 1.09 (− 1.50 to − 0.69)	− 0.098 (− 0.117 to − 0.080)	< 0.001
Sex			
Female	− 1.45 (− 1.68 to − 1.21)	− 0.082 (− 0.104 to − 0.060)	< 0.001
Male	− 0.87 (− 1.53 to − 0.21)	− 0.111 (− 0.139 to − 0.084)	< 0.001
**Comparison between sexes**			
Female	Reference	Reference	–
Male	0.58 (− 0.24 to 1.40)	0.095 (− 1.028 to 1.217)	0.869
p-value	0.1590	–	–
**Length z-score**			
All	− 1.30 (− 1.78 to − 0.83)	− 0.023 (− 0.046 to 0.0001)	0.050
Sex			
Female	− 1.24 (− 2.23 to − 0.25)	− 0.032 (− 0.054 to − 0.010)	0.005
Male	− 1.34 (− 1.92 to − 0.76)	− 0.014 (− 0.050 to 0.023)	0.464
**Comparison between sexes**			
Female	Reference	Reference	–
Male	− 0.11 (− 1.19 to 0.98)	0.689 (− 0.510 to 1.887)	0.260
p-value	0.8365	–	–
**Weight z-score**			
All	− 0.65 (− 0.97 to − 0.33)	− 0.010 (− 0.033 to 0.014)	0.408
Sex			
Female	− 0.49 (− 1.15 to 0.17)	− 0.040 (− 0.067 to − 0.013)	0.004
Male	− 0.75 (− 1.14 to − 0.37)	0.016 (− 0.018 to 0.050)	0.361
**Comparison between sexes**			
Female	Reference	Reference	–
Male	− 0.26 (− 0.98 to 0.46)	0.509 (− 0.550 to 1.567)	0.346
p-value	0.4459	–	–

Of the 23 children with postnatal microcephaly, 15 (65.2%) presented with CNS abnormalities (i.e., ventriculomegaly, cerebral calcifications, cerebellar hypoplasia, trunk hypoplasia, cisterna magna alteration, cortical development disorder, and cortical atrophy) in at least one neuroimaging exam by TFUS, CT, and/or MRI (Table 2 and Supplementary Table 1). Four of the 23 children (17.4%) did not undergo imaging exams (i.e., due to lack of clinical indication), and 4 underwent imaging exams but presented with no abnormalities (one child underwent CT and MRI, two underwent CT and one underwent MRI and TFUS) (Supplementary Table 1). Of the 17 children who underwent CT, 12 (70.6%) presented with imaging abnormalities; of these, 12 (100%) presented with calcifications, 10 (83.3%) with ventriculomegaly and 2 (16.7%) with cortical atrophy. Ten children (43.5%) underwent brain MRIs, and 8 (80.0%) presented with ventriculomegaly. Of the 23 children in the study, 14 (60.9%) presented with craniofacial disproportion, 14 with strabismus, 11 (47.8%) with early onset seizures, 11 (47.8%) with dysphagia, and 10 with arthrogryposis (43.5%) (Table 1 and Supplementary Table 2). With regard to nutritional status at the latest assessment (median 23.3 months, P_25_–P_75_ 18.8 to 27.8), the BMI was within the appropriate parameters for 91.3% of the children. Of the 20 children who underwent fundoscopy and/or RetCam examinations, 8 (40.0%) presented with an abnormality, of which 6 (30.0%) presented with an abnormality in the fundus of the eye, 5 (25.0%) in the optic nerve, and 6 (30.0%) in the retina.

**Table 2:** Clinical (ophthalmologic, neurologic, orthopedic) and brain imaging characteristics of children who developed postnatal microcephaly in the Microcephaly Epidemic Research Group Pediatric Cohort (MERG-PC), in Pernambuco, Brazil.

Eye abnormalities	Frequency among evaluated children	Frequency overall
Strabismus	14/20, 70.0%	14/23, 60.9%
Nystagmus	4/19, 21.1%	4/23, 17.4%
Microphthalmia	1/19, 5.3%	1/23, 4.3%
**Motor and limb abnormalities**		
Arthrogryposis distal	6/19, 31.6%	6/23, 26.1%
Arthrogryposis general	4/19, 21.1%	4/23, 17.4%
Club foot	10/20, 50.0%	10/23, 43.5%
Hip dysplasia	5/19, 26.3%	5/23, 21.7%
**Other affected systems**		
Gastroesophageal reflux	1/7, 14.3%	1/23, 4.3%
Dysmorphic features	20/23, 87.0%	20/23, 87.0%
Craniofacial disproportion	14/19, 73.7%	14/23, 60.9%
Hand contractures	13/19, 6.8%	13/23, 56.5%
**Neurologic abnormalities**		
Epileptic seizures	11/22, 50.0%	11/23, 47.8%
**Brain imaging abnormalities**		
Ventriculomegaly	12/19, 63.2%	12/23, 52.2%
Calcification	12/19, 63.2%	12/23, 52.2%
Cortical atrophy	9/19, 47.4%	9/23, 39.1%
Lissencephaly/pachygyria	3/19, 15.8%	3/23, 13.0%

**Figure 1:**
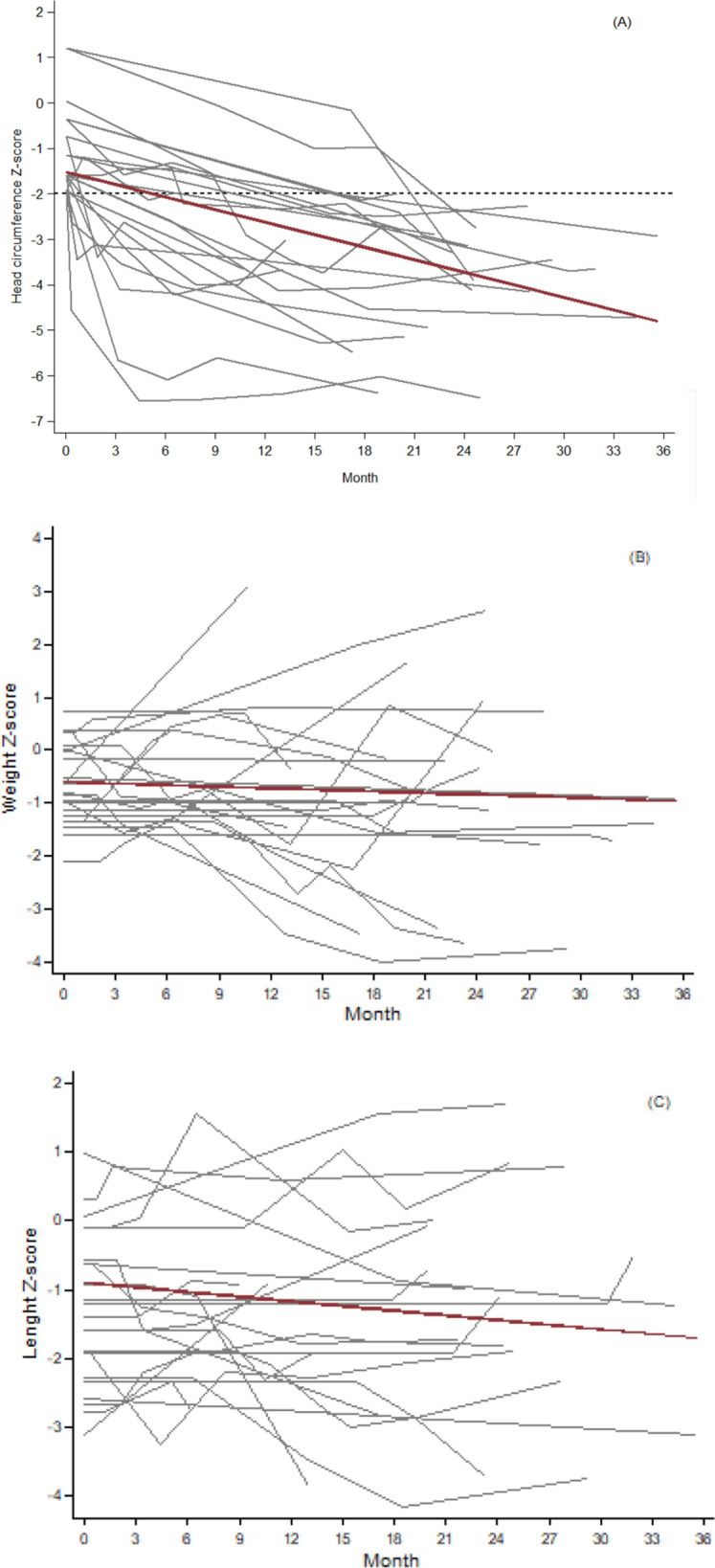
Monthly growth of head circumference (**A**), weight (**B**) and length (**C**) of children who developed postnatal microcephaly in the Pediatric Cohort of the Microcephaly Epidemic Research Group (MERG-PC), in Pernambuco, who developed postnatal microcephaly.

A monthly (i.e., per 30 days) decrease in the HC Z-score (− 0.098 (95% CI − 0.117 to − 0.080, p < 0.001)) was observed both when the children were assessed together and when the assessment was performed separately by sex (male: − 0.111 (− 0.139 to − 0.084); female: − 0.082 (− 0.104 to − 0.060)). The difference between sexes was not statistically significant, p = 0.869 (Table 2 and Fig. 1 (1st graph)). The monthly change in Z-scores for length and weight were, respectively, − 0.023 (95% CI − 0.046 to 0.0001, p = 0.050) and − 0.010 (95% CI − 0.033 to 0.014, p = 0.408) and the difference between sexes was not statistically significant (Table 2 and Fig. 1 (2nd and 3rd graphs)). Most children at the time of the final neurodevelopmental assessment were aged between 7 and 32 months (median, 18 months).

## Discussion

This article has presented a case series of 23 children who presented with a normal Z-score for head circumference at birth and later developed microcephaly. Within this case series, most children presented with severe anatomical lesions in the CNS and almost 50% had ophthalmological abnormalities. The monthly growth velocity of the head circumference in these children, expressed as a Z-score, was − 0.098 (95% CI − 0.117 to − 0.080).

In addition to the report of our case series, postnatal microcephaly related to congenital ZIKV infection has been described elsewhere^22,28,31,32^, although studies were scarce and most refer to a smaller number of cases, ranging between 3 and 13 cases^22,28,31,32^. Only Cavalcante et al.^33^ described a number similar to ours, totaling 28 children. Although the mechanism(s) that leads to postnatal microcephaly are unclear, possible explanations proposed by van der Linden et al.^28^ include the early intrauterine destruction of progenitor cells or other neural cells, the persistence of the inflammatory response or the persistence of the infection in the neural cells. Another factor that may influence the prenatal or postnatal development of microcephaly could be the location of the brain damage. Additional studies are still needed to further clarify this mechanism.

The measurement of HC from 0 to 24 months (i.e., the period with the greatest postnatal growth) is an important anthropometric indicator of brain growth^34,35^ and risks of neurological development deviations. By 12 months of age, the brain will have completed half of its postnatal growth and would be expected to reach 75% of the adult brain size in children without exposure to congenital infections^34,36^. The assessment of head circumference growth using the Z-score enables standardized comparisons that account for age and sex. Published studies that reported postnatal microcephaly have not described the velocity of head circumference growth over time, making it difficult to compare to our results. The speed of decline observed in our case series was lower than that observed in children who already presented with microcephaly at birth, which has been reported as a monthly change in Z-score of − 0.46 in the study by Silva et al.^32^. The difference is likely to be associated with the severity of neurological damage and/or different follow-up times in the two studies. Indeed, when the observation time of the children in our study was restricted to a period of 8 months, similar to Silva et al.^32^, we obtained a similar monthly growth velocity of − 0.32 (95% CI − 0.42 to − 0.21). This follow-up time corresponds to the period in which HC growth is more accelerated in healthy children, which would make the difference more evident between the values observed in these children in relation to the standard for age and sex. Aguiar et al.^37^ followed a group of 87 children with CZS, of whom 77% presented with microcephaly at birth, for three years, a period of follow-up similar to our study and also observed an impairment in the mean HC Z-score during the study period. As the reported HC Z-scores decreased from − 2.75 ± 0.2 SD at birth to − 5.2 ± 0.3 SD between 31 and 36 months, this suggests the children with CZS would have experienced a monthly growth velocity of approximately − 0.07, similar to that observed in the current study. However, this comparison needs to be interpreted with caution as the monthly growth velocity was estimated using only the extreme values, not taking into account variations throughout the observation period, and was based on a sample of children with CZS with and without microcephaly at birth. Further studies with larger sample sizes, such as the planned individual participant data meta-analyses of the Zika Brazilian Cohorts-Consortium^38^ will be necessary to more accurately compare the findings across study sites. Although HC at birth and during development are associated with sex and is lower in female children^35,39,40^, we observed no statistically significant differences between the sexes in terms of growth velocity of HC, suggesting that the mechanism underlying delayed microcephaly is similar in children of both sexes.

The main alterations observed in the neuroimaging exams of the children that made up our case series were reduced brain volume, ventriculomegaly, subcortical calcifications and cortical malformations, similar to that described in the study by Van der Linden et al.^28^. Studies comparing characteristics of microcephaly at birth with postnatal microcephaly^31,33^ have reported differences in the frequency and/or type of structural changes. Aragão et al.^31^ observed cerebral calcifications in the cortex and subcortical white matter in both groups, although cerebral calcifications with another location were observed only in microcephaly at birth. On the other hand, polymicrogyria, possibly a less severe malformation of cortical development, was not detected in the microcephaly group at birth, but was observed in children who presented postnatal microcephaly or did not present with microcephaly. Cavalcante et al.^33^ reported a higher frequency of ventriculomegaly, reduced brain parenchyma, malformation of cortical development and hypoplasia or malformation of the brainstem in children with microcephaly at birth than in the comparison group, which was composed of children without microcephaly at birth but the majority (87.5%) of whom developed postnatal microcephaly. The findings of these studies suggest a greater severity of structural changes in congenital microcephaly than in postnatal microcephaly.

In our study, in addition to structural changes in the central nervous system, a significant percentage of children with postnatal microcephaly also presented ophthalmological abnormalities, other malformations and arthrogryposis, configuring a picture of intense damage caused by ZIKV, similar to that described for cases of microcephaly at birth, and also referred to in the studies by Van der Linden et al.^28^, Cavalcante et al.^33^ and Aguiar et al.^37^.

In our study, the monthly changes in weight and height, expressed in Z-scores were respectively − 0.010 (95% CI − 0.033 to 0.014) and − 0.023 (95% CI − 0.046 to 0.0001) per month, while in children with microcephaly in the study by Silva et al.^32^ these figures were, respectively − 0.08 per month and − 0.16 per month although the data of these authors refer only to the first 8 months of life. Since our results contain zero in the confidence intervals, the data may indicate individual variations in opposite directions over time or that there was no change in the growth rate. For girls as a group, there was a decrease in the Z-score over time, but also with individual variations during the follow-up period.

In summary, the brain damage that led to the development of postnatal microcephaly was not accompanied by a unique pattern of weight and height development. This variability will depend on a number of factors, such as the presence of dysphagia, described in approximately 80% of children with microcephaly^17^, or the presence of endocrine disorders^18^. Cavalcante et al.^33^ reported a small decrease in the mean Z-score of weight for age and length for age, in the period from birth to 36 months, both in the group that presented microcephaly at birth and in that composed mainly of children who developed postnatal microcephaly. The study of Aguiar et al.^37^ also showed a fluctuation in the mean Z-score for weight and length over time among the children with CZS born with and without microcephaly. To detect possible differences between the sexes and between the intensity of brain damage and the evolution of weight and height in children with ZIKV-related microcephaly, studies are needed with a larger sample size that may be achieved in meta-analyses with individual data.

The study presents advantages and limitations. This is one of the few studies that describes cases of postnatal microcephaly and estimate the growth velocity of head circumference, height, and weight during the first months of life. Although children were monitored regularly using standardized procedures and instruments, it remains possible that there was a misclassification bias due as the frequency and timing of measurements performed were not uniform for all children in the study.

## Conclusion

Postnatal microcephaly, which occurs mainly in children who have already presented signs of severe brain damage at birth, is part of the broad spectrum of Congenital Zika Syndrome. This study provides evidence that children who experience postnatal microcephaly present with variability in their weight and height development, which do not manifest as single specific patterns. Although the possible mechanisms underlying the development of postnatal microcephaly still need to be better understood, our findings reinforce the need for strict monitoring of children with prenatal ZIKV exposure by the pediatrician and a multidisciplinary team over the first 1000 days.

## Supplementary Information

Supplementary Tables.

## References

[1] Miranda-Filho DB, Martelli CMT, Ximenes RAA, Araújo TVB, Rocha MAW, Ramos RCF, Dhalia R, França RFO, Marques Júnior ETA, Rodrigues LC (2016). Initial description of the presumed congenital Zika syndrome. Am. J. Public Health.

[2] Del Campo M, Feitosa IML, Ribeiro EM, Horovitz DDG, Pessoa ALS, França GVA, García-Alix A, Doriqui MJR, Wanderley HYC, Sanseverino MVT (2017). The phenotypic spectrum of congenital Zika syndrome. Am. J. Med. Genet. Part A.

[3] Moore CA, Staples JE, Dobyns WB, Pessoa A, Ventura CV, Fonseca EB, Ribeiro EM, Ventura LO, Nogueira-Neto N, Arena JF (2017). Characterizing the pattern of anomalies in congenital Zika syndrome for pediatric clinicians. JAMA Pediatr..

[4] Ventura CV, Maia M, Bravo-Filho V, Góis AL, Belfort R (2016). Zika virus in Brazil and macular atrophy in a child with microcephaly. Lancet.

[5] Van der Linden V, Rolim Filho EL, Lins OG, Van der Linden A, Aragão MFVV, Brainer-Lima AM, Cruz DDCS, Rocha MAW, Silva PFS, Carvalho MDCG (2016). Congenital Zika syndrome with arthrogryposis: Retrospective case series study. BMJ Open.

[6] Leal MC, Muniz LF, Caldas Neto SS, van der Linden V, Ramos RC (2020). Sensorineural hearing loss in a case of congenital Zika virus. Braz. J. Otorhinolaryngol..

[7] Peçanha PM, Júnior SCDSM, Pone SM, Pone MVDS, Vasconcelos Z, Zin A, Vilibor RHH, Costa RP, Meio MDBB, Nielsen-Saines K (2020). Neurodevelopment of children exposed intra-uterus by Zika virus: A case series. PLoS ONE.

[8] Faiçal AV, De Oliveira JC, Oliveira JVV, De Almeida BL, Agra IA, Alcantara LCJ, Acosta AX, De Siqueira IC (2019). Neurodevelopmental delay in normocephalic children with in utero exposure to Zika virus. BMJ Paediatr. Open.

[9] Gerzson LR, De Almeida CS, Da Silva JH, Feitosa MMA, De Oliveira LN, Schüler-Faccini L (2019). Neurodevelopment of nonmicrocephalic children, after 18 months of life, exposed prenatally to Zika virus. J. Child Neurol..

[10] Cranston JS, Tiene SF, Nielsen-Saines K, Vasconcelos Z, Pone MV, Pone S, Zin A, Salles TS, Pereira JP, Orofino D (2020). Association between antenatal exposure to Zika virus and anatomical and neurodevelopmental abnormalities in children. JAMA Netw. Open.

[11] Van der Linden V, van der Linden Junior H, Leal MC, Rolim Filho EL, Linden AVD, Aragão MFVV, Brainer-Lima AM, Cruz DDCS, Ventura L, Florêncio TLT (2017). Discordant clinical outcomes of congenital Zika virus infection in twin pregnancies. Arq. Neuropsiquiatr..

[12] World Health Organization. *Child Growth Standards* (WHO, 2006) https://www.who.int/tools/child-growth-standards (Accessed 5 Dec 2020).

[13] Brasil. Ministério da Saúde. Secretaria de Atenção à Saúde. Diretrizes de estimulação precoce: crianças de zero a 3 anos com atraso no desenvolvimento neuropsicomotor decorrente de microcefalia (Ministério da Saúde, 2016).

[14] Chan JFW, Choi GKY, Yip CCY, Cheng VCC, Yuen KY (2016). Zika fever and congenital Zika syndrome: An unexpected emerging arboviral disease. J. Infect..

[15] Adair LS, Fall CH, Osmond C, Stein AD, Martorell R, Ramirez-Zea M, Sachdev HS, Dahly DL, Bas I, Norris SA (2013). Associations of linear growth and relative weight gain during early life with adult health and human capital in countries of low and middle income: Findings from five birth cohort studies. Lancet.

[16] Soares F, Abranches AD, Villela L, Lara S, Araújo D, Nehab S, Silva L, Amaral Y, Junior SCG, Pone S, Lobkowicz L, Clemente NS, Brasil P, Nielsen-Saines K, Pone M, Brickley E, Moreira ME (2019). Zika virus infection in pregnancy and infant growth, body composition in the first three months of life: A cohort study. Sci. Rep..

[17] Oliveira DMS, Miranda-Filho DB, Ximenes RAA, Montarroyos UR, Martelli CMT, Brickley EB, Gouveia MCL, Ramos RC, Rocha MAW, Araujo TVB (2020). Comparison of oropharyngeal dysphagia in Brazilian children with prenatal exposure to Zika virus, with and without microcephaly. Dysphagia.

[18] Veras Gonçalves A, Miranda-Filho DD, Rocha Vilela LC, Ramos RCF, de Araújo TV, de Vasconcelos RAL, Wanderley Rocha MA, Eickmann SH, Cordeiro MT, de Oliveira Ventura MLV (2020). Endocrine dysfunction in children with Zika-Related microcephaly who were born during the 2015 epidemic in the state of Pernambuco, Brazil. Viruses.

[19] INTERGROWTH-21st. http://intergrowth21.ndog.ox.ac.uk/ (Accessed 5 Dec 2020).

[20] Miranda-Filho DB, Brickley EB, Ramond A, Martelli CMT, Clemente NS, Araújo TVB, Rodrigues LC, Montarroyos UR, de Souza WV, de Albuquerque MFPM (2021). On Behalf Of The Microcephaly Epidemic Research Group. The Microcephaly Epidemic Research Group Paediatric Cohort (MERG-PC): A cohort profile. Viruses.

[21] Curvas de Referência da Organização Mundial da Saúde. https://www.sbp.com.br/departamentos-cientificos/endocrinologia/graficos-de-crescimento/ (Accessed 15 Aug 2021).

[22] Franca GV, Schuler-Faccini L, Oliveira WK, Henriques CM, Carmo EH, Pedi VD, Nunes ML, Castro MF, Serruya S, Silveira MF (2016). Congenital Zika virus syndrome in Brazil: A case series of the first 1501 livebirths with complete investigation. Lancet.

[23] Brasil. Protocolo de Vigilância e Resposta à Ocorrência de Microcefalia Relacionada à Infecção pelo Vírus Zika–Plano Nacional de Enfretamento à Microcefalia (Ministério da Saúde, 2015).

[24] World Health Organization, Anthro for personal computers, version 3.2.2, 2011: Software for assessing growth and development of the world's children. (WHO, 2010) http://www.who.int/childgrowth/software/en/ (Accessed 22 June 2019).

[25] Ashwal S, Michelson D, Plawner L, Dobyns WB (2009). Practice parameter: Evaluation of the child with microcephaly (an evidence-based review). Report of the Quality Standards Subcommittee of the American Academy of Neurology and the Practice Committee of the Child Neurology Society. Neurology.

[26] Mlakar J, Korva M, Tul N, Popović M, Poljšak-Prijatelj M, Mraz J (2016). Zika virus associated with microcephaly. N. Engl. J. Med..

[27] Hazin AN, Poretti A, Martelli CMT, Huisman TA (2016). Computed tomographic findings in microcephaly associated with Zika virus. N. Engl. J. Med..

[28] Van der Linden V, Pessoa A, Dobyns W, Barkovich AJ, Júnior HV, Filho EL, Ribeiro EM, Leal MC, Coimbra PP, Aragão MF (2016). Description of 13 infants born during October 2015–January 2016 with congenital zika virus infection without microcephaly at birth—Brazil. MMWR Morb. Mortal. Wkly. Rep..

[29] World Health Organization. Anthro for Personal Computers, Version 3.2.2, 2011: Software for Assessing Growth and Development of the World’s Children (WHO, 2010) http://www.who.int/childgrowth/software/en/ (Accessed 22 June 2019).

[30] Puska, P., Nishida, C. & Porter, D. Obesity and overweight. Charts and tables:child gowth standards for children aged under 5 years. (World Health Organization, 2003) https://www.who.int/en/news-room/fact-sheets/detail/obesity-and-overweight (Accessed 22 June 2019).

[31] Aragao MFVV, Holanda AC, Brainer-Lima AM, Petribu NCL, Castillo M, van der Linden V, Serpa SC, Tenório AG, Travassos PTC, Cordeiro MT (2017). Nonmicrocephalic infants with congenital zika syndrome suspected only after neuroimaging evaluation compared with those with microcephaly at birth and postnatally: How large is the zika virus "iceberg"?. AJNR. Am. J. Neuroradiol..

[32] Silva AAM, Ganz JS, Sousa PD, Doriqui MJ, Ribeiro MR, Branco MD, Queiroz RC, Pacheco MJ, Costa FRV, Silva FS (2016). Early growth and neurologic outcomes of infants with probable congenital zika virus syndrome. Emerg. Infect. Dis..

[33] Cavalcante TB, Ribeiro MRC, Sousa PS, Costa EPF, Alves MTSSB, Simões VMF, Batista RFL, Takahasia EHM, Amaral GA, Khourif R (2021). Congenital Zika syndrome: Growth, clinical, and motor development outcomes up to 36 months of age and differences according to microcephaly at birth. Int. J. Infect. Dis..

[34] Biller J, Gruener G, Brazis P (2011). DeMyer’s The Neurologic Examination: A Programmed Text.

[35] Macchiaverni LM, Barros Filho AA (1998). Perímetro Cefálico: Por que medir sempre. Medicina.

[36] Graber, E.G. Crescimento físico de lactentes e crianças (Sydney Kimmel Medical College, 2019) https://www.msdmanuals.com/pt/profissional/pediatria/crescimento-e-desenvolvimento/introdu%C3%A7%C3%A3o-ao-crescimento-e-desenvolvimentosionais (msdmanuals.com).

[37] Aguiar EBD, Pone SM, Gomes Junior SCDS, Soares FVM, Zin AA, Vasconcelos ZFM, Ribeiro CTM, Pereira Junior JP, Moreira MEL, Nielsen-Saines K (2022). Anthropometric parameters of children with congenital zika virus exposure in the first three years of life. Viruses.

[38] Alecrim MDGC, Amorim MMR, Araújo TVB, Brasil P, Brickley EB, Castilho MDC, Coelho BP, Cunha AJLAD, Duarte G, Estofolete CF, Gurgel RQ, Herrero-Silva J, Hofer CB, Lopes ASA, Martelli CMT, Melo ASO, Miranda-Filho DB, Montarroyos UR, Moreira ME, Mussi-Pinhata MM, Oliveira CS, Passos SD, Prata-Barbosa A, Santos DND, Schuler-Faccini L, Silva AAMD, Siqueira IC, Sousa PDS, Turchi MD, Ximenes RAA, Zara ALSA, Zika Brazilian Cohorts Consortium Zbc-Consortium (2021). Zika Brazilian Cohorts (ZBC) Consortium: Protocol for an individual participant data meta-analysis of congenital zika syndrome after maternal exposure during pregnancy. Viruses.

[39] Araújo CLP, Dutra CLC, Hallal PC (2007). Validity of maternal report on birth weight 11 years after delivery: The 1993 Pelotas Birth Cohort Study, Rio Grande do Sul State, Brazil. Cad. Saúde Pública.

[40] Jaldin MGM, Pinheiro FSP, Santos AM, Muniz NC, Brito LMO (2011). Head circumference growth of exclusively breastfed infants during the first six months of life. Rev. Pau. Pediatr..

