# Supplementary material for: Characteristics of children of the Microcephaly Epidemic Research Group Pediatric Cohort who developed postnatal microcephaly
Source: Sci Rep. 2022 Sep 22;12:15778. doi: 10.1038/s41598-022-19389-w (PMC9500100; doi:10.1038/s41598-022-19389-w)
Supplement: Supplementary file 1 — Supplementary Tables. [file 41598_2022_19389_MOESM1_ESM.docx]

**Supplementary Table 1- Biological and anthropometric characteristics of children who developed postnatal microcephaly in the Microcephaly Epidemic Research Group Pediatric Cohort (MERG-PC), in Pernambuco, Brazil.**

| **Patient** | **Sex** | **Birth Gestational age (wks)** | **Maternal Zika virus** | **Infant Zika virus IgM antibody by ELISA** | **At Birth** | | | | **SGA fetus at birth (Y/N)** | **Last visit** | | | | | **Imaging** | **Degree of evidence CZS** |
| --- | --- | --- | --- | --- | --- | --- | --- | --- | --- | --- | --- | --- | --- | --- | --- | --- |
|  |  |  |  |  | **HC (cm)** | **HC (Z-Score)** | **Weight (g)** | **Length (cm)** |  | **HC (cm)** | **HC (Z-Score)** | **Age (months)** | **Weight**  **(grams)** | **Length**  **(centimeters)** |  |  |
| 1 | M | 40 |  | negative (CSF) | 32 | -1.94 | 3236 | 40.3 | No | 39.5 | -6.49 | 24.9 | 12,295 | 82 | CT abnormal/MRI abnormal | Confirmed |
| 2 | F | 40 |  | positive (CSF) | 32 | -1.59 | 4110 | 49 | No | 40.2 | -3.67 | 13.1 | 7,130 | 69.5 | CT abnormal | Confirmed |
| 3 | F | 41 | positive (IgM) |  | 32 | -1.59 | 2245 | 44 | Yes | 41.5 | -4.11 | 24.4 | 9,560 | 80.9 | CT normal | Probable |
| 4 | M | 41 |  | positive (CSF) | 32 | -1.94 | 3650 | 50 | No | 43 | -4.14 | 27.9 | 14,100 | 93 | CT abnormal/MRI abnormal | Confirmed |
| 5 | M | 38 |  | positive (CSF) | 32 | -1.94 | 2665 | 44 | Yes | 41 | -2.01 | 6.20 | 8,300 | 62 | No | Confirmed |
| 6 | M | 41 | positive (IgM) |  | 36 | 1.21 | 3600 | 52 | No | 43 | -3.90 | 24.5 | 16,600 | 92.8 | No | Probable |
| 7 | M | 38 |  |  | 34 | -0.36 | 2900 | 47 | No | 44 | -3.14 | 24.2 | 11,715 | 84 | CT normal/ MRI normal | Possible |
| 8 | F | 39 |  |  | 32 | -1.59 | 2995 | 44.5 | No | 43 | -3.45 | 29.2 | 8,000 | 77 | CT abnormal/  TS-US normal | Confirmed |
| 9 | M | 40 |  | positive (CSF) | 33.5 | -0.76 | 3840 | 88.8 | No | 40 | -5.48 | 17.3 | 7,300 | 74 | CT abnormal/  TS-US normal | Confirmed |
| 10 | M | 40 |  | negative (CSF) | 34.5 | 0.03 | 3476 | 47 | No | 43.7 | -3.28 | 23.3 | 7,880 | 76 | CT normal/MRI abnormal | Confirmed |
| 11 | F | 40 |  | positive (CSF) | 32 | -1.59 | 3585 | 48 | No | 40 | -4.93 | 21.8 | 7,400 | 79 | CT abnormal/  TF-US normal | Confirmed |
| 12 | M | 38 |  | positive (CSF) | 33 | -1.15 | 2960 | 48 | No | 44 | -1.59 | 31.9 | 11,145 | 91.5 | No | Confirmed |
| 13 | F | 40 |  |  | 32 | -1.59 | 3350 | 52 | No | 39.5 | -5.14 | 20.3 | 9,750 | 83 | CT abnormal | Confirmed |
| 14 | M | No information |  | positive (CSF) | 32 | -1.94 | 3100 | 50 | No | 39 | -6.38 | 18.8 | 10,900 | No  information | CT abnormal | Confirmed |
| 15 | M | 39 | positive (PCR) |  | 34 | -0.36 | 2785 | 47 | No | 44 | -2.76 | 20.0 | 10,480 | 84 | TF-US  abnormal/ CT normal | Confirmed |
| 16 | F | 38 |  |  | 33 | -0.74 | 3190 | 49.5 | No | 44.5 | -2.27 | 27.8 | 9,900 | 81 | CT and MRI abnormal | Confirmed |
| 17 | M | 39 |  |  | 32 | -1.94 | 2820 | 47 | No | 42.5 | -3.01 | 13.2 | 9,528 | 72.5 | CT and MRI abnormal | Confirmed |
| 18 | M | No  Information |  |  | 34 | -0.36 | 3300 | 48 | No | 34 | -2.00 | 19.9 | 10,200 | 82.1 | No | Probable |
| 19 | M | No information |  | negative (CSF) | 36 | 1.21 | 3830 | 48 | No | 44.6 | -2.74 | 24.7 | 10,800 | 90.3 | MRI and  TF-US  normal | Probable |
| 20 | F | 39 |  | positive (CSF) | 32 | 0.99 | 3230 | 51 | No | 41.7 | -1.22 | 34.3 | 10,780 | 89.3 | CT and MRI abnormal | Confirmed |
| 21 | M | 39 |  |  | 32 | -3.11 | 3050 | 44 | No | 41 | -0.91 | 10.7 | 13,040 | 72 | CT normal/  MRI abnormal | Confirmed |
| 22 | F | 40 |  |  | 32 | -1.15 | 3050 | 47 | No | 43 | -2.92 | 35.6 | 8,808 | 83.7 | MRI abnormal | Confirmed |
| 23 | F | 40 |  |  | 32 | -0.62 | 3160 | 48 | No | 42.9 | -0.98 | 22.2 | 10,800 | 81.7 | TF-US and CT abnormal | Confirmed |

**Supplementary Table 2 – Length of follow-up and clinical characteristics of children who developed postnatal microcephaly in the Pediatric Cohort of the Microcephaly Epidemic Research Group (MERG-PC), in Pernambuco, who developed postnatal microcephaly.**

| **Patient** | **Follow-up (months)** | **Sex** | **Malformation** | **Craniofacial disproportion** | **Arthrogryposis** | **Hip dysplasia** | **Epileptic seizures** | **Club foot** | **Strabismus** | **Nystagmus** | **Dysphagia** |
| --- | --- | --- | --- | --- | --- | --- | --- | --- | --- | --- | --- |
| 1 | 24,9 | M | yes | yes | yes | no | No | yes | yes | no | Yes |
| 2 | 13,1 | F | yes | yes | no | no | yes | yes | yes | no | No |
| 3 | 24,4 | F | no | N.A. | N.A. | N.A. | no | N.A. | N.A. | N.A. | No |
| 4 | 27,9 | M | yes | yes | no | no | yes | yes | yes | no | Yes |
| 5 | 6,2 | M | yes | no | no | no | no | no | no | no | Yes |
| 6 | 24,5 | M | no | N.A. | N.A. | N.A. | N.A. | N.A. | N.A. | N.A. | N.A. |
| 7 | 24,2 | M | yes | yes | no | no | no | no | yes | no | Yes |
| 8 | 29,2 | F | yes | no | no | no | no | yes | no | no | Yes |
| 9 | 17,3 | M | yes | N.I. | N. I. | N.I. | yes | yes | yes | N. I. | Yes |
| 10 | 23,3 | M | yes | yes | yes | no | yes | no | yes | yes | Yes |
| 11 | 21,8 | F | yes | yes | yes | no | yes | yes | yes | yes | Yes |
| 12 | 31,9 | M | yes | yes | yes | yes | no | no | no | no | No |
| 13 | 20,3 | F | yes | yes | yes | yes | yes | yes | yes | no | Yes |
| 14 | 18,8 | M | yes | yes | no | no | no | no | yes | yes | No |
| 15 | 20,0 | M | yes | no | no | no | no | no | no | no | No |
| 16 | 27,8 | F | no | N.A. | N.A. | N.A. | no | N.A. | N.A. | N.A. | No |
| 17 | 13,2 | M | yes | yes | no | no | no | no | yes | no | No |
| 18 | 19,9 | M | no | no | yes | no | no | yes | no | no | N.A. |
| 19 | 24,7 | M | yes | no | no | no | yes | no | yes | no | No |
| 20 | 34,3 | F | yes | yes | yes | yes | yes | yes | yes | no | No |
| 21 | 10,7 | M | yes | yes | yes | no | yes | no | yes | no | Yes |
| 22 | 35,6 | F | yes | yes | yes | yes | yes | yes | yes | no | No |
| 23 | 22,2 | F | yes | yes | Yes | no | yes | no | no | no | Yes |
